# Improving Burial Practices and Cemetery Management During an Ebola Virus Disease Epidemic — Sierra Leone, 2014

**Published:** 2015-01-16

**Authors:** Carrie F. Nielsen, Sarah Kidd, Ansumana R.M. Sillah, Edward Davis, Jonathan Mermin, Peter H. Kilmarx

**Affiliations:** 1 Center for Global Health, CDC; 2 National Center for HIV/AIDS, Viral Hepatitis, STD, and TB Prevention, CDC; 3 Sierra Leone Ministry of Health and Sanitation; 4 Department for International Development, United Kingdom

As of January 3, 2015, Ebola virus disease (Ebola) has killed more than 2,500 persons in Sierra Leone since the epidemic began there in May 2014 (1). Ebola virus is transmitted principally by direct physical contact with an infected person or their body fluids during the later stages of illness or after death (2). Contact with the bodies and fluids of persons who have died of Ebola is especially common in West Africa, where family and community members often touch and wash the body of the deceased in preparation for funerals. These cultural practices have been a route of Ebola transmission (3). In September 2014, CDC, in collaboration with the Sierra Leone Ministry of Health and Sanitation (MOH), assessed burial practices, cemetery management, and adherence to practices recommended to reduce the risk for Ebola virus transmission. The assessment was conducted by directly observing burials and cemetery operations in three high-incidence districts. In addition, a community assessment was conducted to assess the acceptability to the population of safe, nontraditional burial practices and cemetery management intended to reduce the risk for Ebola virus transmission. This report summarizes the results of these assessments, which found that 1) there were not enough burial teams to manage the number of reported deaths, 2) Ebola surveillance, swab collection, and burial team responses to a dead body alert were not coordinated, 3) systematic procedures for testing and reporting of Ebola laboratory results for dead bodies were lacking, 4) cemetery space and management were inadequate, and 5) safe burial practices, as initially implemented, were not well accepted by communities. These findings were used to inform the development of a national standard operating procedure (SOP) for safe, dignified medical burials, released on October 1. A second, national-level, assessment was conducted during October 10–15 to assess burial team practices and training and resource needs for SOP implementation across all 14 districts in Sierra Leone. The national-level assessment confirmed that burial practices, challenges, and needs at the national level were similar to those found during the assessment conducted in the three districts. Recommendations based on the assessments included 1) district-level trainings on the components of the SOP and 2) rapid deployment across the 14 districts of additional trained burial teams supplied with adequate personal protective equipment (PPE), other equipment (e.g., chlorine, chlorine sprayers, body bags, and shovels), and vehicles. Although these assessments were conducted very early on in the response, during October–December national implementation of the SOP and recommendations might have made dignified burial safer and increased community support for these practices; an evaluation of this observation is planned.

During an Ebola epidemic, prevention and control measures include prompt and safe burial of the dead (2). A safe burial can be accomplished by a trained burial team using appropriate PPE, placing the body in a puncture- and leak-resistant plastic body bag, and burying the body in a grave at least 2 meters deep (4,5). Ideally, used burial team PPE should be incinerated or buried at a depth of at least 2 meters (4,5). During July and August, MOH-supported waste management and burial trainings were conducted for newly established burial teams. Burial teams that were added after these trainings were trained individually within their respective districts. The initial MOH protocol included the deployment of three different functional teams after a reported death. These teams were 1) a case investigation (Ebola surveillance) team to collect risk factor and contact information, 2) an oral swab–collection team for Ebola testing, and 3) an eight to 12-person burial team. In September, in response to growing evidence of substantial Ebola virus transmission associated with funeral attendance and contact with the bodies of Ebola victims, CDC and the MOH conducted assessments of burial practices in preparation for developing and disseminating guidance and the SOP.

Sierra Leone, in West Africa, is divided into 14 districts (Figure 1). The capital of Sierra Leone, Freetown (estimated population of 1 million), is in Western Urban District. In September 2014, five direct observational assessments were conducted in Western Urban, Western Rural, and Port Loko districts, areas with high Ebola incidence (Figure 1). Practices of burial teams, swab-collection teams, cemetery management teams, and cemeteries were directly observed and recorded. The observers conducted the direct observations without wearing PPE but remained at a safe distance of at least 3 meters away from the body at all times. In the two Western districts, the two cemeteries designated for Ebola victims were visited and assessed (King Tom for Western Urban and Waterloo for Western Rural). In Port Loko District, a cemetery designated for Ebola victims, as well as community burials (burials conducted by burial teams using single graves dug by the community outside of an established cemetery) were evaluated. In addition, a community assessment of the acceptability of safe burial practices was conducted in Western Urban, Western Rural, and Port Loko districts. While the burial teams were removing the bodies, 15 short interviews were conducted with community counselors and family and community members that were near the homes where the person had died to learn the community and family perceptions of the cause of death, burial team procedures, and Ebola in the community.

On October 1, an SOP for safe, dignified medical burials was approved and released by the Sierra Leone National Emergency Operations Center (4). During October 10–15, a national-level rapid needs assessment was conducted to assess the national-level burial team practices and determine the training and resource needs for effective implementation of this SOP. In all, 12 of 14 MOH district burial team supervisors and one district medical officer were interviewed about trainings received, the process for coordinating supplies, and logistics for the daily activities of the burial teams, as well as burial team composition, practices, challenges, and needs (Table).

## Burial Team Assessments — September and October 2014

Before October 2014, the national safe burial system in Sierra Leone required substantial human resources and was logistically complicated, resulting in delays in collecting bodies. As observed during this study, after a death, it could take 1–5 days to pick up the body. The coordination of the case investigation, swab collection, and burial teams was inconsistent, and burial teams often arrived at the location of a deceased person and buried the body before a swab specimen was collected or a case investigation was completed. When a burial team was in a community, a community counselor or leader would directly inform the team of other deceased persons requiring pick-up, which the burial team would then collect. This would bypass the official reporting structure and further complicate the coordination of investigation and swab-collection teams. Burial team size varied from six to 12 persons. Where security was a concern, a police escort sometimes accompanied the burial team.

Nationally, burial team supervisors received reports about dead bodies from many different sources and requested improved coordination and communication in their respective districts when receiving notification of a death. Notification occurred through various channels, including the district hotline or alert system, the district surveillance teams, and direct calls from community members. Whether a death was officially recorded depended on how it was reported. Deaths were not recorded if they were reported directly by community members rather than through the official alert system, leading to underreporting of deaths. For example, of the 12 bodies that were collected over a 1-day period by two Port Loko burial teams, five deaths were reported directly to the burial team supervisor by a community counselor while the burial teams were out picking up other bodies. Because these five deaths were not reported through the designated alert channels, they were not officially recorded, representing an undercount of 42% for that day for the two teams, although this is based on very small numbers.

**FIGURE 1:**
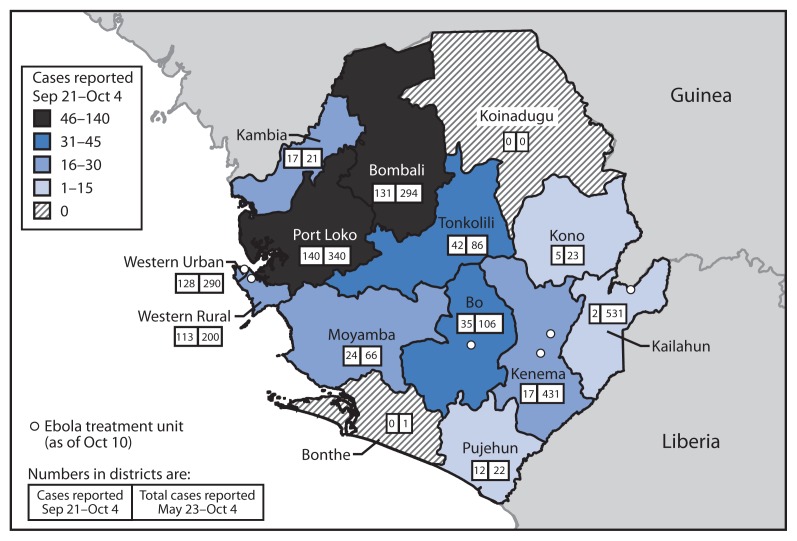
Cumulative number of confirmed Ebola virus disease (Ebola) cases, by district — Sierra Leone, September 21–October 4, 2014 Sources: Sierra Leone Ministry of Health and Sanitation and International Federation of Red Cross and Red Crescent Societies.

**FIGURE 2:**
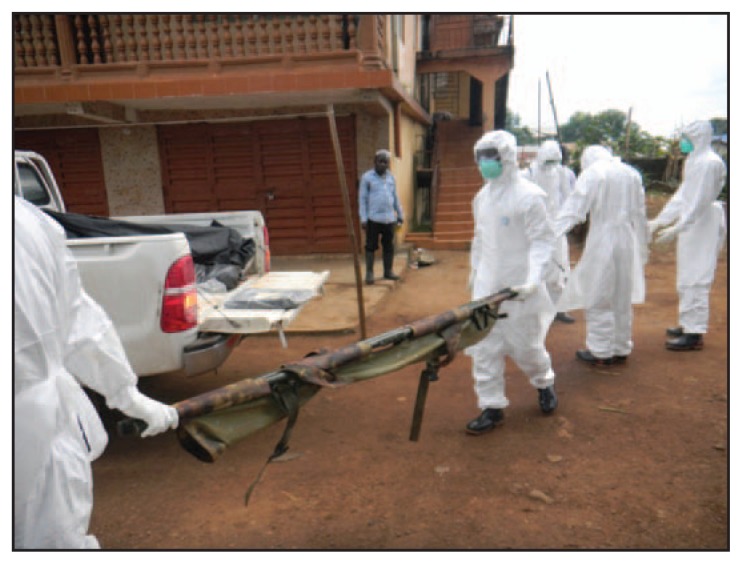
A burial team preparing to collect another body and transport it in the back of truck along with eight other bodies that had already been collected — Sierra Leone, September 2014

The number of deaths to which the teams responded on a daily or weekly basis varied greatly (Table). The number of bodies that teams collected was typically higher in districts that had an Ebola treatment center or large holding facility, because the number of Ebola patients (and deaths) was higher in a facility than in the general community (Table and Figure 1). Burial teams collected as many as 10 bodies each day, which required each team member to don and doff up to 11 sets of PPE each day (the last set of PPE is donned when the burial team arrives at the cemetery) (Figure 2). This frequent change of PPE was required for safety, but could also provide occasions for breaches in infection prevention and control, specifically breaches in stringent procedures for PPE use. It also created a substantial volume of hazardous medical waste to be disposed of.

During observations, burial teams performed most infection prevention and control practices well, including using PPE and chlorine for disinfection. However, one major concern was that after body removal, decontamination of homes was limited to spraying all surfaces with 0.5% hypochlorite solution (undiluted household bleach). Bedding and other potentially contaminated materials were not removed, despite a recommendation to dispose of them (4,5).

During direct observation of burial teams over 3 days in the two Western districts, the homes of only two of 22 persons who had died had been visited by a case management team before the burial team arrived to remove the body. In addition, of 22 bodies collected by burial teams, a swab specimen was collected from only three. The burial team supervisor had to explain to these 19 families that they could either keep the body in a separate room and wait for a swab team to arrive and collect a specimen, then wait for the test result before learning if they could bury the body, or the burial team could remove the body without a test result. By the time the burial team arrived, often more than 24 hours had elapsed since the person had died. All 19 families chose not to wait for a swab specimen to be collected but to have the body removed for safe burial.

## Cemetery and Burial Practices Assessments — September and October 2014

**FIGURE 3:**
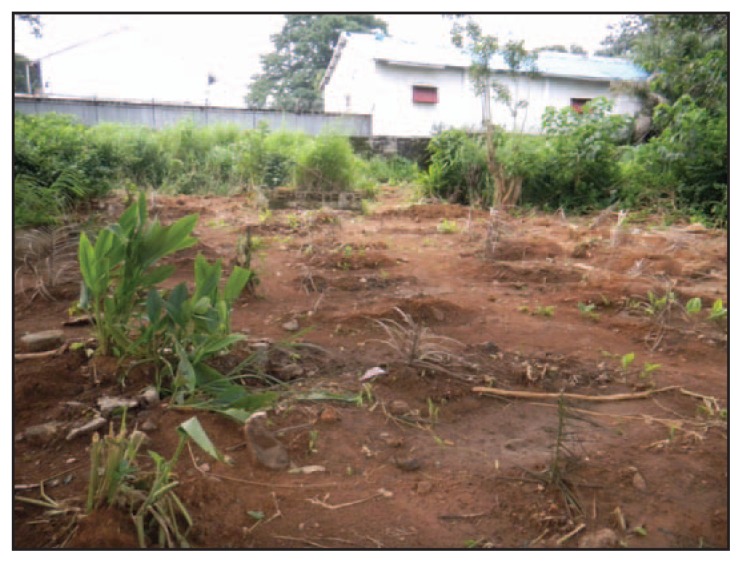
Unmarked graves in an Ebola burial section of a cemetery — Sierra Leone, September 2014

**FIGURE 4:**
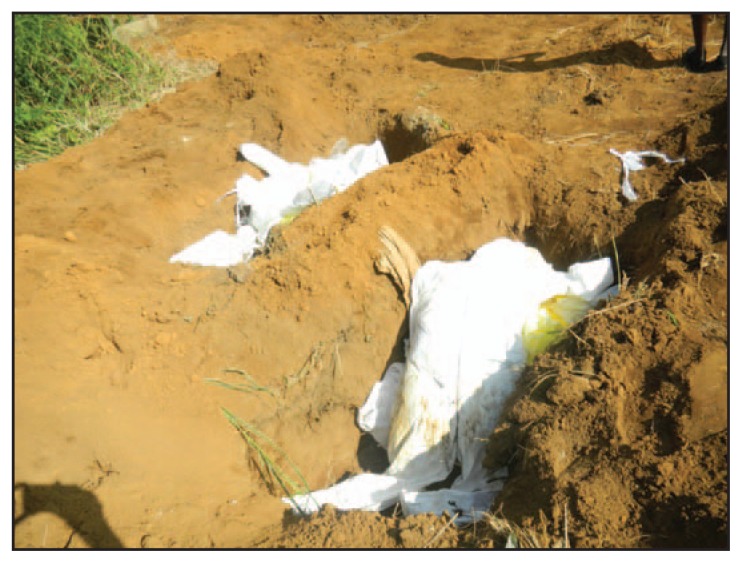
Dead bodies, personal protective equipment, and medical waste buried together in unmarked graves at an unsafe depth of <2 meters — Sierra Leone, September 2014

In Western Urban District, King Tom Cemetery was the only approved location for safe burials. By October 7, a total of 420 bodies had been buried there; during September to mid-October, 30–40 bodies were buried each day. At the time of the assessment, the cemetery was not fenced and pedestrian traffic through the cemetery was high. Unmarked graves were hand-dug (Figure 3), often not to the recommended depth, and were too few to accommodate all the bodies brought to the cemetery each day. More than one body was sometimes placed into a single grave (Figure 4). Because of the challenge of appropriately disposing of large volumes of used PPE, it was placed on top of bodies, nearly filling the graves. Thus, bodies and medical waste were often not buried as deep as 2 meters, the recommended safe depth (4,5). Cemetery managers did not allow family members in the cemetery, and family members were not able to observe their loved ones’ burial. Funerals for persons who had not been tested for Ebola were still being performed in the non-Ebola areas of the cemetery by burial agencies or mortuaries.

Safe burials in Western Rural and Port Loko districts included both cemetery and community burials. Both districts had a designated cemetery, which was an isolated open area of unfenced land where graves were dug. Families were not allowed to observe cemetery burials. Safe community burials were taking place with the approval of the chiefdom leader, which permitted the community of the person who had died to dig a grave in an area that was agreed upon by the family and the community. Then the burial team would place the body in the body bag and bury the body in the grave, usually while the community looked on.

Bodies were buried, not cremated. Districts with larger cities or towns were more likely to bury bodies in cemeteries (Table and Figure 1). Each day, burial teams collected all bodies reported to them before going to the cemetery. This often resulted in many bodies being placed together in the backs of trucks in unmarked body bags (Figure 2), which prevented individual identification and distressed the families.

## Community Assessment — September 2014

Fifteen community and family interviews were conducted. An important concern among them was that family members were being buried in unmarked graves, often with multiple bodies in the same grave. These practices were considered undignified and unacceptable by the community. Safe community burials were more acceptable to community members than safe cemetery burials because families were more involved and procedures were more transparent. In addition, for 11 of 12 bodies collected by burial teams being observed in Western Urban District, the community counselors that were interviewed reported that the cause of death was unknown, and that there were no known suspected or confirmed Ebola cases in the community. During the interviews of community and family members, it was also learned that many were not aware of the risk for Ebola transmission from contact with an infectious dead body, many denied that Ebola was real, witchcraft was reported to be the cause of one death, and in one instance, an “attack” from something other than Ebola was reported as the cause.

What is already known on this topic?

Ebola virus can be transmitted through exposure to the body of an Ebola patient who has recently died, which can occur during funeral ceremonies in which mourners have direct contact with the body.

What is added by this report?

In September 2014, CDC, in collaboration with the Sierra Leone Ministry of Health and Sanitation, assessed burial practices, cemetery management, and adherence to practices recommended to reduce the risk for Ebola virus transmission in three districts with a high-incidence of Ebola virus disease (Ebola). In addition, a community assessment was conducted to assess the acceptability to the population of changes in burial practices and cemetery management intended to reduce the risk for Ebola transmission. It was found that 1) there were not enough burial teams to manage the number of reported deaths, 2) Ebola surveillance, Ebola swab collection for postmortem testing, and burial team responses to a dead body alert were not coordinated, 3) systematic procedures for testing and reporting of Ebola laboratory results for dead bodies were lacking, 4) cemetery space and management were inadequate, and 5) safe burial practices, as initially implemented, were not well accepted by communities.

What are the implications for public health practice?

Since the assessments, there have been many improvements, nationally, in safe and dignified burial practices in Sierra Leone. Fully implementing a standard operating procedure for safe, dignified medical burial nationally might decrease further transmission of Ebola virus in the country.

## Recommendations and Development of Standard Operating Procedures

Recommendations based on these findings from the September assessment included 1) developing multifunction burial teams that are trained to complete case investigation forms and collect swab specimens from dead bodies, and 2) conducting safe and dignified medical burials. To improve community acceptance of safe burials, increased community outreach and cemetery improvements were recommended, such as removing waste, adding fencing, and marking graves. These findings were used in developing the national SOP for safe, dignified medical burials. The SOP, with special consideration of burial practices consistent with a person’s religious faith, was developed and approved by the Sierra Leone Emergency Operations Center on October 1 (4). The primary purpose of the SOP was to provide operational guidance for the classification of deaths, proper burial in a safe and dignified manner, and the disposal of potentially contaminated materials from the household when the body was collected. The SOP included guidance that during the epidemic, in high-incidence areas, all deceased persons should be buried by the burial teams within 24 hours, irrespective of laboratory results. Because of limited laboratory capacity, the guidance recommends swabbing only bodies of suspected and probable Ebola victims and the bodies of persons who died from an unknown cause. Deaths clearly attributable to another cause or of previously confirmed Ebola victims did not need swab testing. The policy could be further refined to address the management of dead bodies in areas with less Ebola virus transmission.

As the epidemic continues, coordination of safe burial teams is important, and district-level coordination is needed in each of Sierra Leone’s 14 districts. To address the scale of the epidemic and ensure that each burial team collected a manageable number of bodies each day (judged to be five or fewer) and donned and doffed PPE a reasonable number of times per day, it was recommended that the number of burial teams in the country be increased to 120. Engagement with religious and traditional leaders to build important alliances within the community also was recommended to encourage community acceptance of safe, dignified medical burials.

Given the size of the ongoing epidemic, additional Ebola deaths are expected, and additional cemeteries for Ebola burials should be designated. In addition, as specified in the SOP, safe community burials by burial teams should be allowed, when space is available.

## National Response — October–December 2014

**FIGURE 5:**
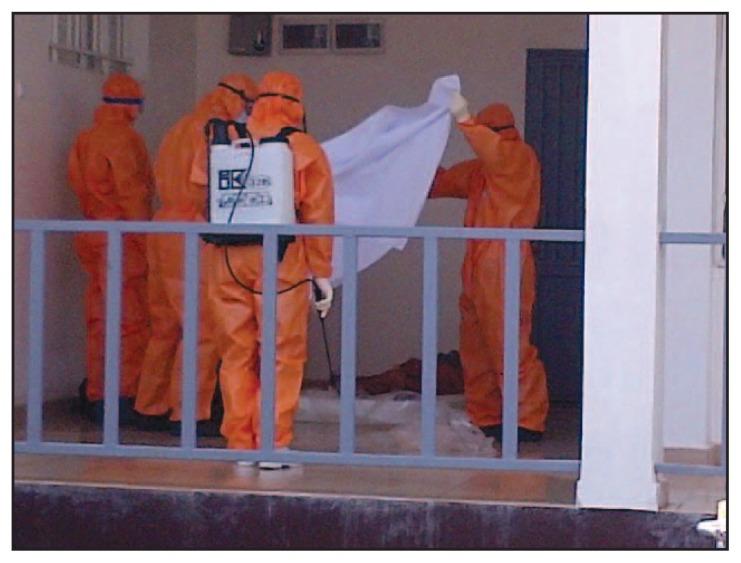
A burial team preparing to wrap a body in a Muslim shroud, illustrating the incorporation of a dignified component of a standard operating procedure for safe, dignified medical burial — Sierra Leone, October 2014

In October, a major focus of national social mobilization efforts was the “Safe Burials Save Lives” campaign. The campaign spread the message that dead bodies needed to be handled with extreme caution and that safe burials prevent Ebola virus transmission. It promoted the SOP for safe, dignified medical burials. On October 18, 149 out of 150 Paramount Chiefs (nonpartisan members of Parliament in the government of Sierra Leone) and other traditional leaders met to discuss the need for safe burials and recognized the need to provide leadership to improve community acceptance of safe burials. Ongoing efforts to incorporate some traditions into safe burial practices continues, including the use of shrouds for Muslim families and the use of coffins for families that provide them (Figure 5). Additional safe and dignified burial practices have been implemented allowing the community and family to honor and respect the deceased; these practices include 1) allowing families to provide special clothing to the burial team to dress the deceased before they are placed inside the body bag, 2) allowing the families to come to the cemeteries to observe the burial, and allowing them to invite an imam or minister to pray with the families at a safe distance, and 3) allowing, when possible, burial teams to conduct safe community burials close to the home of the deceased.

On October 19, with significant support from the government of the United Kingdom, a burial team command center was launched for Western Rural and Western Urban districts. This center, staffed by Republic of Sierra Leone Armed Forces troops, was responsible for coordinating the activities of all burial teams, including 1) dispatching teams, swab collection coordination, and managing cemeteries; 2) determining in real-time which burial team would respond to each reported death; and 3) ensuring that records related to each body were managed appropriately. Changes in burial team structures also were initiated to include a team of newly trained persons, with one person designated to fill out the case investigation form and another person trained to collect swab specimens. These additional persons were dispatched at the same time as the burial teams. All burial teams also were restructured to include at least one supervisor to coordinate with the identified community counselor or leader and family members, and supervise the activities of their burial team. By October 31, there were 70 trained burial teams, nationally. By the end of November, safe burial command centers were established in eight additional districts (Port Loko, Bombali, Moyamba, Kambia, Tonkolili, Kenema, Koinadugu, and Bo).

In response to the findings of inadequate Ebola cemetery management and in anticipation of future burial capacity needs in King Tom Cemetery in Freetown, major improvements of the cemetery were initiated. Improvements included removing waste, constructing a perimeter wall, marking and recording grave sites, and allowing families to visit the cemetery and observe safe burials. As of October 26, a total of 891 bodies had been buried in King Tom Cemetery.

### Discussion

These preliminary assessments of burial practices in Sierra Leone found that 1) deaths were not always reported or not recorded when reported directly to the burial teams, 2) testing of bodies for Ebola was not always performed in situations where it was recommended, 3) decontamination of homes where Ebola deaths had occurred was often incomplete, and 4) not all bodies were collected by burial teams. Numerous examples of bodies being handled in an inappropriate and undignified manner were identified and included unmarked body bags being loaded into a truck, bodies being placed in unmarked graves, failure to respect or follow religious practices, and family members not being allowed to observe the burial of a loved one. By discouraging reporting and proper burial of bodies, these problems might have contributed to ongoing Ebola virus transmission in Sierra Leone. In response to these and other assessments, guidance and a safe and dignified burial SOP were developed and are being implemented across the country and have led to burying bodies in a safer, yet dignified, manner that allows many religious and cultural traditions to be honored. Ongoing efforts in Sierra Leone have addressed all of the recommendations in this report. It will be important to document these dramatic improvements.

The findings in this report are subject to at least two limitations. First, the findings of the assessment are based on relatively small sample sizes of direct observations and unstructured interviews of available family and community members. Second, the national level burial team supervisor assessment was conducted soon after the approval of the SOP and before the rapid scale-up of burial teams and command centers, which has since improved burial practices. Nonetheless, this is the first documentation of challenges faced by communities and authorities trying to make burials safe and dignified during the Ebola epidemic in Sierra Leone.

There have been challenges to changing behaviors related to safe burials, with continuing reports of unsafe and secret burials leading to new Ebola outbreaks in Sierra Leone (6). Continuing to improve the dignity of safe burial process by treating all bodies with respect, observing customs and religious practices to the extent that they do not endanger the living, and allowing community involvement to occur at safe distances, as called for in the SOP, might increase the acceptability of safe burials. This would likely reduce Ebola virus transmission because deaths would be more likely to be reported and bodies more likely to be buried safely by burial teams.

An important lesson from the first large-scale Ebola epidemic is the need for plans to effectively and safely handle the bodies of persons who have died from Ebola, and to execute these plans in a dignified and respectful manner that honors the deceased, their families, and their communities. Rapidly scaling up of safe, dignified burial practices and focusing on increasing community acceptance of safe burials during an Ebola epidemic could interrupt transmission substantially (7). Since the time of these assessments, considerable improvements have been made in burials nationally.

## Figures and Tables

**TABLE:** Burial team supervisors’ assessments on burial team composition, practices, challenges, and needs, by district — Sierra Leone, October 1–10, 2014

District	No. of trained burial teams	No. of persons per team	No. of functional burial teams	How being alerted to a request for a body collection	Average body collection per day	Bodies collected within 24 hours	Cemetery or community burials	Swabs performed by laboratory technician	Challenges and needs
Kambia	1	8 (includes grave digger)	1	Receiving calls from community and holding center	1–2	Yes	Both	No swabs	Would like 1–2 more teams and a coordinator
Port Loko	7 (5 are emergency responder teams, district is split into 2 zones; 3 chiefdoms per team)	7 (only 2 supervisors)	7	Through hotline	15	Mostly, except when vehicle challenges occur	Cemetery for bodies collected from health facilities and community burials; district-level grave digger stays at cemetery	Just started October 1	Just received WHO vehicles but might not be sufficient for rough roads; also only 3 of the teams are being paid regularly
Bombali	2 (3rd team being trained)	11	2	Getting called directly by community and by surveillance team	10–15	80%	Cemetery 2–3 miles from Makeni and community	Swab team just started on October 2	Getting support from WHO but still need strong vehicles
Koinadugu	1 (2nd team being trained)	Team 1 = 8 (includes grave digger)	1	Called by surveillance team	2 bodies total		Community	Yes; both bodies were swabbed and negative	Getting 2 more vehicles next week, waiting for 2nd team to start after training
Tonkolili	1	8	11 (disagreement on recognition of burial teams)	Most bodies are from holding center, not community	3 (12 bodies buried in total)	Not all	Cemetery	Not coordinated	Additional teams are working and being paid, but only one has been trained by MOH and it is the only one the supervisor recognizes; need vehicles and more compensation for cemetery land owners
Kono	Cannot be reached								
Kenema	4	6 (no supervisor)	3	By holding center or surveillance team	6–7 from holding center, 4 from community; has decreased since treatment center moved	1–3 days; road access challenges cause delays	All bodies from treatment center buried in Red Cross Cemetery; other cemetery full	Yes; either laboratory technician goes with or they bring body to mortuary, but do not wait for results	Need more vehicles and sometimes the surveillance team uses their sprayers for their surveillance team visits
Kailahun	2 IFRC 4 MOH	8	2	By DMO and MSF	≥200 bodies buried to date		Cemetery	Technician just started following IFRC burial teams	Improve communication and coordination between DMO and IFRC
Bo	4	8	4	Surveillance team	4	1–3 days	Cemetery	Yes	Doing OK since WHO sent vehicles; gaining community confidence with swabs
	Training 5th team this week	Includes grave digger		Getting 4–7 calls per day				Since swabs introduced 2 weeks ago (September 22)	
Pujehun	2, as per the DMO	8; includes a grave digger			8 confirmed deaths (unknown); often do not have a body to collect in a given week				Would like more vehicles that can handle rough terrain, fuel, and refresher training
Bonthe	2	8	1; only have 1 vehicle to get burial team to site, but then they do not transport body anywhere; it is carried to the community site	Do not have a hotline, community leaders call DHMT	1–2 per week; only 2 bodies collected total	Delays because of road conditions	Community	Sometimes laboratory technician shows up to body	Need vehicles appropriate for body transport and need new spraying equipment, theirs keeps breaking
Moyamba	14	8	2	Getting called directly by community and by hotline	3–4 per day	1–2 days because distance to get to bodies is far	Both	Only bodies in Moyamba town are swabbed	Would like support for at least 3 more teams, would like vehicles that can handle difficult terrain, fuel; also rainy season is now so would like rain gear
	1 trained per chiefdom	Includes grave digger	MOH only paying 2 of the teams		From community and holding center			Additional burial team members being trained to collect swabs, October 9	
Western Area	11	12	10	Hotline and called directly	30–40; approximately 20 buried in King Tom Cemetery daily	No	Cemetery	Some still waiting for results before burying body	Need more burial teams, vehicles, and improved communication and coordination

**Abbreviations:** WHO = World Health Organization; MOH = Ministry of Health and Sanitation, Sierra Leone; DMO = District Medical Officer; MSF = Médecins Sans Frontières; IFRC = International Federation of Red Cross and Red Crescent Societies; DHMT = District Health Management Team.
